# Development and psychometric evaluation of the type 1 diabetes mellitus self-management scale for parents

**DOI:** 10.1007/s00431-024-05650-z

**Published:** 2024-06-12

**Authors:** Merve Aşkın Ceran, Murat Bektaş, Beray Selver Eklioğlu

**Affiliations:** 1 Department of Pediatric Nursing, Institute of Health Sciences, Dokuz Eylul University, İzmir, Türkiye; 2 KTO Karatay University, Vocational School of Health Services, Konya, Türkiye; 3 Department of of Pediatric Nursing, Dokuz Eylul University, Faculty of Nursing, İzmir, Türkiye; 4 Division of Pediatric Endocrinology, Necmettin Erbakan University Faculty of Medicine, Konya, Türkiye

**Keywords:** Parents, Type 1 diabetes mellitus, Self-management, Psychometric, Development, Scale

## Abstract

**Background/aim:**

Diabetes has become a global epidemic, necessitating effective self-management strategies. This is particularly crucial for parents of children with type 1 diabetes mellitus, as they must make numerous daily decisions and perform complex care activities. Therefore, the aim of this study was to develop a comprehensive diabetes self-management scale specifically for parents of children with type 1 diabetes. This scale aims to holistically address behaviors impacting diabetes self-management and to evaluate its psychometric properties.

**Materials and methods:**

A methodological, correlational, and cross-sectional study was conducted with a sample of 190 parents of children with type 1 diabetes mellitus. The scale items were reviewed by five experts to ensure they adequately covered the parents' evaluation of their children's diabetes self-management. Following this, a Turkish language expert assessed the draft scale for language accuracy, comprehensibility, and grammar. The data were analyzed using descriptive statistics (numbers and percentages), Cronbach's α reliability coefficient, factor analysis, and correlation analysis.

**Results:**

The Cronbach's alpha for the overall scale was 0.893, and the Cronbach's alpha for the subscales was between 0.757 and 0.845. The item-total score correlations ranged between 0.408 and 0.660 (*p* < .05). The exploratory factor analysis showed that the scale explained 61.427% of the total variance, and the factor loadings of items ranged from 0.574 to 0.859. The confirmatory factor analysis also showed that the factor loadings of the scale items ranged from 0.574 to 0.859.

*Conclusion*: The validity and reliability analyses revealed that the scale is a valid and reliable measurement tool for the Turkish culture.

## Introduction

Type 1 Diabetes Mellitus (T1DM) is a chronic disease that presents with high blood sugar resulting from insufficient secretion of insulin due to the destruction of beta cells in the pancreas. It requires long-term care and causes changes in the lifestyle of the individual and the family [1]. The incidence of T1DM varies worldwide due to both genetic and environmental factors. According to the International Diabetes Federation (IDF), more than 1.2 million children and adolescents under the age of 20 have T1DM worldwide [2]. It is reported that the rate of development of T1DM in children under the age of 18 in Turkey is 10.8/100.000 a year and the incidence has increased over the years [3].

Individuals with a chronic disease such as T1DM need continuous medication, regular treatment, or the use of special medical equipment to survive. T1DM care needs of children in the age of growth and development are different and more complex than adults. The main difficulties in meeting these needs arise from factors such as the child's psychological characteristics, health status, family dynamics, and the need for care outside the home (e.g., at school) [4]. As the treatment of T1DM is complex and multidimensional, children and adolescents may sometimes have difficulties in diabetes self-management. The diagnosis of T1DM affects the family negatively as well as the child. Families may experience physical and functional losses with the diagnosis of a chronic disease. They may also lose hopes and dreams about their child, the family identity they had before the illness, and their freedom due to added responsibilities. The chronic illness may become the center of family life, which may lead to conflicts between the child and his family from time to time [5, 6] and which may require the family to make many daily self-management decisions and perform complex care activities [7].

Self-management is essential for the successful management of T1DM. Significant improvements can be achieved in HbA1c level, self-efficacy goals, stress management, and self-management behaviors such as support and decision making through the improvement of self-management skills. Self-management includes personalized diet and exercise programs in addition to tight blood sugar control to prevent acute and chronic complications that may occur in diabetic individuals. It also facilitates the evaluation and management of lifelong psychosocial issues [8]. Additionally, social support plays a significant role in managing chronic diseases. Family members, in particular, create an environment that encourages self-care behaviors, such as adherence to dietary guidelines and regular physical activity. When necessary, they can help children develop self-care behaviors and increase their motivation by providing feedback [9]. It is important to provide children with T1DM regular diabetes education based on family cooperation and to help them acquire problem-solving skills in order to ensure successful diabetes management [10, 11].

There is a need for programs that ensure the continuity of behavioral strategy development, incorporate social support, and enhance metabolic recovery for effective diabetes self-management [12]. Given that diabetes has become a global issue, effective self-management is essential. Therefore, studies should focus on developing scales to evaluate diabetes self-management specifically for parents who provide primary care to children with diabetes, thereby increasing the effectiveness of interventions. While there are studies on diabetes self-management and related scales in Turkey, they often focus on specific aspects of diabetes mellitus [13–15] and are generally designed for adolescent and adult populations. Currently, there is no scale to evaluate diabetes self-management for parents, which results in parents of children with type 1 diabetes mellitus being unable to adequately assess their self-management levels.

The present study differs from previous studies by adopting a holistic approach to the behaviors that affect diabetes self-management. It aims to develop a self-management scale specifically for parents of children aged 7–12 years with type 1 diabetes and to evaluate its psychometric properties.

## Design and method

A methodological, correlational and cross-sectional study was designed to develop the Type 1 Diabetes Mellitus Self-Management Scale for Parents (T1DMS-P) and to determine its psychometric properties.

### Sample

The sample for this study consisted of parents of children aged 7–12 years with T1DM who visited the pediatric endocrine outpatient clinic at a university hospital. Data were obtained from 190 parents who agreed to participate in the study, whose child had been diagnosed with T1DM for at least six months, and who had no chronic disease or communication disability.

In validity and reliability studies, it is recommended to recruit 3, 5, or 10 participants per item on the scale. If the number of items in the scale is small, a minimum of 100 participants is suggested [16]. For the 47-item Diabetes Self-Management Scale, a minimum sample size of 141 parents (3 participants per item) was required. A total of 190 parents who met the research criteria were included in the sample to ensure a more representative sample and enhance the statistical power of the analysis.

### Scale development

The items and subscales of the scale, developed to assess the self-management skills of parents of children with diabetes, were determined based on a comprehensive review of the literature. An item pool was created by reviewing the studies and scales in the literature on self-management, self-management in diabetes, factors affecting childhood diabetes management, and the risk factors that affect blood sugar level and the management of complications [13–15, 17]. In addition, diabetes care standards and childhood diagnosis and treatment guidelines of institutions such as the World Health Organization, Turkish Endocrinology and Metabolism Association, and American Diabetes Association were examined [18, 19]. As a result of the literature review, a pool of 47 items was created.

### Content validity

The opinions of five experts—a pediatric endocrinologist, three pediatric nursing experts specializing in diabetes, and a public health nursing expert—were solicited to ensure the scale items adequately reflected parents' views on their children's T1DM self-management. Using the Davis technique, the experts assessed the items based on content and age-group suitability, rating them on a four-point scale: (a) appropriate, (b) item should be revised, (c) item should be seriously revised, and (d) item is not appropriate. The Content Validity Index (CVI) for each item and the overall scale was calculated by dividing the number of experts who selected options (a) and (b) by the total number of experts. Based on expert feedback, one item was removed, resulting in a 46-item scale. The CVI for individual items ranged from 0.98 to 1.00, and the overall scale's CVI was 0.99. The draft scale was then reviewed by a Turkish language expert for language accuracy, comprehensibility, and grammar. Following these evaluations, the draft scale was deemed suitable for piloting.

### Pilot study

It is recommended in the literature that draft scales should be administered to a group of 20–30 people who have similar characteristics to the sample and that the participants of a pilot study should not be included in the research sample [20–22]. In the pilot study, the 46-item scale was administered to 20 parents of children with diabetes. With the pilot test, the readability, intelligibility and response time of the scale were evaluated. Parents completed the scale in an average of 15 min. No negative feedback was received from the parents about the readability and intelligibility of the scale. The pilot study showed that the items of the T1DM Self-management Scale for Parents were easy to understand. The data obtained from the pilot study and the participants of the pilot study were not included in the validity and reliability study.

### Data collection method

Data were collected from the parents who were admitted to the endocrine outpatient clinic and who agreed to participate in the study and sign the consent form (n = 190). The Sociodemographic Data Collection Form and the T1DM Self-Management Scale for Parents (T1DMS-P) were utilized to collect data.

### Sociodemographic data collection form

The form was developed by the researchers. It consists of 9 items to determine the sociodemographic characteristics of the parents of children with diabetes (age, gender, degree of closeness to the child, educational status, income level, working status, marital status, the person monitoring the child for diabetes, and place of residence).

### Type 1 diabetes mellitus self-management scale for parents (T1DMS-P)

Following validity and reliability analyses, the T1DM Self-Management Scale for Parents (T1DMS-P) underwent refinement from its original 46 items to a concise set of 15 items distributed across three key sub-dimensions. The Cronbach's Alpha coefficients demonstrated strong internal consistency, with values of 0.889 for both the "blood glucose management" and "health check-up" sub-dimensions, and 0.868 for the "exercise and cooperation" sub-dimension. The overall Cronbach's Alpha for the scale was calculated at 0.935, indicating high reliability.

Each item on the scale is assessed using a five-point Likert scale, ranging from 1 (strongly disagree) to 5 (strongly agree). The scoring system allocates a minimum of 5 points and a maximum of 25 points to the "blood glucose management" sub-dimension, a minimum of 4 points and a maximum of 20 points to the "health check-up" sub-dimension, and a minimum of 6 points and a maximum of 30 points to the "exercise and cooperation" sub-dimension. Thus, the total score that could be obtained from the scale ranges between 15 and 75, with higher scores reflecting greater levels of diabetes self-management among parents of children with diabetes.

There is no predefined cut-off point within the scale, and all items are positively coded, without any reverse-coded items. This scale is designed specifically for parents of children aged 7–12 years who have been diagnosed with type 1 diabetes mellitus at least six months prior to assessment.

### Data analysis

In this study, a combination of statistical software programs was employed for comprehensive analysis. SPSS 24.0 was utilized for descriptive statistics, including calculations of numbers, percentages, and means. Additionally, SPSS was used to conduct Cronbach's alpha analysis, item-total score analysis, Pearson correlation analysis, Split-half analysis, and Hotelling T-square test [33] for evaluating response bias.

The Jamovi program, version 2.2, was employed to calculate the McDonald omega coefficient, which provides an alternative measure of internal consistency reliability.

For assessing content validity, the Content Validity Index (CVI) was computed. Both Exploratory Factor Analysis (EFA) and Confirmatory Factor Analysis (CFA) were conducted to evaluate the construct validity of the scale. In the EFA, Principal Axis Factoring with Promax rotation was used to determine subscales, with an eigenvalue threshold of 1 utilized for subscale determination. A factor loading coefficient of 0.32 was considered to decide item placement under specific subscales. The EFA utilized a correlation matrix.

The CFA, conducted using the AMOS 24.0 program, confirmed the structure identified in the EFA. Before performing the CFA, normality and multicollinearity analyses were conducted to ensure data integrity. Convergent and discriminant validity analyses based on the CFA results were also carried out. Throughout the analyses, a margin of error of *p* = 0.05 was maintained to ensure statistical significance.

### Ethical considerations

This research complies with the principles outlined in the Declaration of Helsinki. Ethics committee approval was obtained from the Non-Pharmaceutical and Non-Medical Device Research Ethics Committee (Decision number 2021/010) of a university to conduct the study. Written approval (no: E-14567952–900-147026) was obtained from the chief physician of the hospital where the study was conducted.

## Results

### Demographic data

The mean age of the parents was found to be 38.58 ± 6.466. 60% of the participants live in the city and 40% live in the countryside. 77.9% of the parents are female and 22.1% are male. Of the parents in the study, 76.9% are mothers, 22.1% are fathers and 1% grandmothers. 8.7% are literate, 41.5% are primary school graduates, 25.6% are high school graduates, 19.5% have a two-year college degree, and 9 have a bachelor’s degree. 31.3% of the parents stated that their income is less than their expenses, while 59% reported that their income is equal to their expenses and 9.7% stated that their income is more than their expenses. It was seen that 96.8% of the parents have been working, 96.9% are married, and mostly mothers (83.1%) follow the child's diabetes.

### Preliminary analyses

In the first stage, the EFA was performed and 21 items with a cumulative value of less than 0.50 and a factor loading less than 0.30 were removed from the scale. Within the scope of the CFA, 10 items with factor loadings below 0.30, CR < 0.70 and AVE < 0.50 were removed from the scale as a result of the convergent and discriminant validity analysis. Analyses were carried out with the remaining 15 items (Table 1).

**Table 1:** Results of the Exploratory Factor Analysis (*n* = 190)

Items	Factor Loads		
	First Subscale	Second Subscale	Third Subscale
I1 (DSMS-P 1)	0.576		
I2 (DSMS-P 7)	0.831		
I3 (DSMS-P 8)	0.730		
I4 (DSMS-P 10)	0.843		
I5 (DSMS-P 12)	0.824		
I6 (DSMS-P 17)		0.601	
I7 (DSMS-P 18)		0.697	
I8 (DSMS-P 26)		0.729	
I9 (DSMS-P 27)			0.699
I10 (DSMS-P 28)			0.859
I11 (DSMS-P 30)			0.738
I12 (DSMS-P 33)			0.730
I13 (DSMS-P 36)		0.756	
I14 (DSMS-P 41)		0.684	
I15 (DSMS-P 45)		0.574	
Eigenvalue	8.070	1.188	1.085
Explained Variance (%)	51.351	5.461	4.616
Explained Total Variance (%)	61.427		
KMO	0.925		
Bartlett X^2^(p)	1870.716 (***p* < 0,001**)		

*DSMS-P*, Development Self-Management Scale- Parents, *KMO*, Kaiser-Mayer-Olkin

### Exploratory factor analysis

In the second stage, the EFA was performed again. The Kaiser-Meyer Olkin (KMO) coefficient was found to be 0.925, and Bartlett’s test result was X^2^ = 1870.716 (*p* < 0.001). The EFA revealed that the scale consisted of three subscales with an eigenvalue greater than 1.00. The scale was found to explain 61.427% of the total variance. The first subscale explained 51.351% of the total variance; the second subscale explained 5.461% of the total variance, and the third subscale explained 4.616% of the total variance. It was determined that the factor loadings of the first subscale varied between 0.576–0.843, while those of the second and third subscales varied between 0.574–0.756 and 0.699–0.859, respectively (Table 1).

### Confirmatory factor analysis

**Fig. 1:**
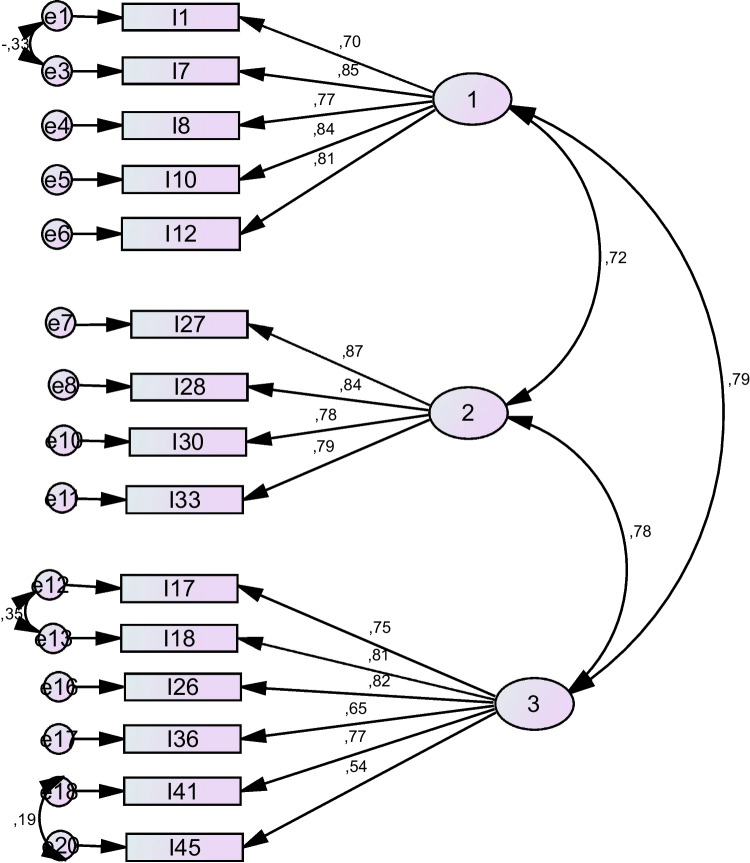
Confirmatory Factor Analysis of the Diabetes Self-Management Scale for Parents (*n* = 190)

The CFA revealed the factor loadings of the subscales as follows: the first subscale 0.576–0.843; the second subscale 0.574–0.756, and the third subscale 0.699–0.859 (Fig. 1). The Chi-square of the three-factor model was 146.720; the degree of freedom was 84; and p < 0.001. The X^2^/SD was found to be 1.747. The fit indices (GFI, CFI, NFI and IFI) were found to be greater than 0.90, and RMSEA was found to be less than 0.080 (Table 2).

**Table 2:** Confirmatory Factor Analysis Model Fit Indices (*n* = 190)

	X^2^	DF^a^	X^2^/SD	RMSEA^b^	GFI^c^	CFI^d^	IFI^e^	TLI^f^	NFI^g^
Third Factor Model	146.720	84	1.747	0.063	0.911	0.966	0.966	0.957	0.924

a = Degree of Freedom, b = Root Mean Square Error of Approximation, c = Goodness of Fit İndex, d = Comparative Fit İndex, e = İncremental Fit İndex, f = Tucker–Lewis İndex, g = Normed Fit Index

In the comparison of the upper-lower group of 27%, the mean score of the upper group was 69.92 ± 3.78 and that of the lower group was 40.46 ± 9.55 (t = 20.257, *p* < 0.05).

According to the CFA analysis results, the convergent and discriminant validity of the scale was examined. It was found that the CR value was greater than 0.70, the AVE value was above 0.50, and CR > AVE in four subscales. These results showed that the scale had convergent validity. As a result of the analysis, MSV < AVE, ASV < AVE, and the square root of AVE was greater than the correlation between subscales. These results showed that the scale had discriminant validity (Table 3).

**Table 3:** Convergent and Discriminant Validity Results (*n* = 190)

Subscale	CR	AVE	MSV	MaxR(H)	1	2	3	ASV
First subscale	0.897	0.637	0.624	0.904	**0.798**			0.380
Second subscale	0.890	0.670	0.612	0.896	0.725***	**0.819**		0.340
Third subscale	0.871	0.533	0.624	0.887	0.790***	0.415***	**0.730**	0.300

*** *p* < 0.001, *CR* = Composite Reliability, *AVE* = Average Variance Extracted, *MSV* = Maximum Squared Variance, *ASV* = Average Shared Squered Variance

### Reliability analysis

The Cronbach's alpha of the scale was found to be 0.935. The Cronbach's alpha of the subscales was as follows: the first subscale 0.889, the second subscale 0.889, and the third subscale 0.868. The split-half analysis showed that the Cronbach’s alpha of the first half and the second half was 0.893 and 0.849, respectively. The correlation between the two halves was found to be 0.915. The Spearman-Brown coefficient was calculated as 0.955, and the Guttman split-half coefficient was calculated as 0.935. The McDonald’s Omega coefficient was 0.895 for the whole scale, 0.852 for the first subscale, 0.820 for the second subscale, and 0.817 for the third subscale (Table 4). The Hotelling T-square test was performed to determine whether there was response bias in the scale, and the Hotelling T-square value was found to be 180.121, F = 58.937, and *p* < 0.001. Thus, the analysis revealed that there was no response bias in the scale.

**Table 4:** Reliability Analysis Results (*n* = 190)

			Split Half Analysis					
	Cronbach’s Αlpha	McDonald Omege	Part 1 Cronbach’s Αlpha	Part 2 Cronbach’s Αlpha	Spearman-Brown	Guttman split half	Correlation Between Forms	X ± SD
Scale Total	0.935	0.895	0.893	0.849	0.955	0.953	0.915	55.915 ± 12.25
First subscale	0.889	0.852						19.447 ± 4.407
Second subscale	0.889	0.820						15.247 ± 4.01
Third subscale	0.868	0.817						21.221 ± 5.40

It was found that the correlations of the scale items with the scale total score ranged between 0.500 and 0.761. The correlation of the scale items with the subscale total scores ranged between 0.330 and 0.699 (Table 5). It was determined that there was no item that significantly increased the Cronbach's alpha when removed from the scale.

**Table 5:** Cronbach's Alpha, Item-Total Score, and Item-Subscale Score Correlations when an Item is Deleted (*n* = 190)

ITEMS	Cronbach’s Alpha If Item Deleted	Corrected Item-Total Correlation (r)*	Corrected Item-Subscale Total Score Correlation (r)*
I1 (I think that my child has sufficient knowledge about Diabetes)	0.611	0.508	0.933
I2 (My child can give insulin injections as taught)	0.707	0.690	0.930
I3 (When my child takes his insulin, he knows that he will do it in a different place each time)	0.685	0.614	0.931
I4 (My child adjusts the insulin dose according to the blood glucose result as recommended to him/her)	0.734	0.685	0.929
I5 (My child knows what to do when there are problems with the glucometer he/she uses)	0.699	0.621	0.930
I6 (My child exercises regularly, as recommended by the diabetes team, to prevent blood sugar spikes)	0.714	0.623	0.930
I7 (My child adjusts exercise regimen according to blood glucose results)	0.755	0.678	0.929
I8 (My child always checks his/her blood sugar before starting exercise)	0.735	0.646	0.929
I9 (My child regularly checks his/her feet (cuts, scrapes…))	0.761	0.699	0.928
I10 (My child keeps toenails straight and short)	0.693	0.665	0.930
I11 (My child prefers shoes that do not squeeze their feet)	0.683	0.609	0.931
I12 (My child knows that he/she should have regular eye examinations every year)	0.674	0.595	0.931
I13 (My child reads when given a booklet or a brochure about diabetes)	0.571	0.461	0.934
I14 (My child can achieve their goals to keep their diabetes under control)	0.727	0.615	0.930
I15 (My child participates in diabetes support groups to exchange information about diabetes)	0.500	0.330	0.937

* Significant at *p* < 0.001, *I* = Item

## Discussion

This section discusses the validity and reliability of the T1DM Self-Management Scale for Parents. The scale was presented to five experts to test content validity. Both I-CVI and S-CVI values should be greater than 0.80 to ensure that there is an agreement between expert opinions [23, 24]. In this study, both item-based and scale-based content validity indices were greater than 0.80, which indicated that the scale assessed the construct sufficiently, and content validity was ensured. Content validity analysis showed that the items in the scale were sufficient and appropriate to evaluate the self-management of children with T1DM from parents' perspective [23, 24].

Following the content validity analysis, the construct validity of the scale was first evaluated with the EFA. It was examined whether the sample size was sufficient for the EFA and whether there was a moderate correlation between the items. The adequacy of the sample was evaluated with the KMO value, and the Bartlett test was performed to see if there was a moderate correlation within the dataset. According to the literature, the Bartlett test value should be statistically significant and the KMO value should be at least 0.60 in order to perform factor analysis [20, 21]. In this study, the Bartlett test result was found to be significant and the KMO value was found to be greater than 0.60. These results showed that the sample was adequate, and the correlation matrix was suitable for factor analysis.

Three subscales with eigenvalues greater than 1 were determined in the EFA. Thus, it was decided that the scale would consist of three subscales. The three-factor scale explained 61.427% of the total variance. The literature suggests that the variance explained in new multi-factor scales should be greater than 50% [20, 21]. It is highlighted that higher total variances indicate stronger construct validity [25, 26]. It was determined that the total variance obtained in this study was greater than 50% and the scale had a total variance explained above the recommended level. These results indicate that the scale has construct validity. The EFA revealed that the factor loadings of the items under three subscales ranged from 0.574 to 0.859. The literature suggests that items with a factor loading above 0.30 should be included in scales. In this study, the factor loadings of the items were greater than 0.30.

These results showed that the scale has a strong factor structure [20, 21]. The analysis showed that the items in the scale can reveal the self-management levels of parents of children with T1DM and sufficiently measure the level of self-management and self-efficacy. The EFA revealed that the scale comprises three sub-dimensions: blood sugar management, health monitoring, and exercise and cooperation. These dimensions are critical areas in diabetes self-management, and the scale has been shown to effectively evaluate and measure them from the parent's perspective. Developing a scale that successfully determines the self-management levels of children with T1DM according to these sub-dimensions, especially from the parents' viewpoint, is expected to make a valuable contribution to the literature.

The CFA was performed to confirm the relationship between the items and the subscales and among the subscales obtained as a result of the EFA. The CFA revealed that the ratio of Chi-square value to degrees of freedom was less than five. Factor loadings in all sub-dimensions were found to be greater than 0.30; fit indexes (GFI, CFI, NFI and IFI) were greater than 0.90, and the RMSEA was less than 0.080. A strong and significant relationship was found between the scale and its subscales. In the literature, model fit indices > 0.90, X2/DF value less than five, and an RMSA value < 0.08 are accepted as good fit indicators [27, 28]. The CFA results in this study are consistent with the values stated in the literature. The results revealed that the data were compatible with the model; the structure determined by the EFA was confirmed; the subscales were compatible with the scale, and the items were adequately related to the subscales. The CFA showed that the items could adequately measure what the subscales intend to measure and that they could successfully measure self-management. The CFA supported the conclusion that the sub-dimensions identified in the EFA blood sugar regulation, health monitoring, exercise, and cooperation were compatible with each other and that the scale was sufficient to measure the intended concept. The CFA demonstrated that the items in the scale could adequately assess children's T1DM self-management from a parent's perspective and that the items and subscales were interrelated, confirming the scale's validity and reliability.

The convergent and discriminant validity of the scale was also examined. The CFA showed that the CR value was greater than 0.70, the AVE value was above 0.50, and CR > AVE in all subscales. These results revealed that the scale had convergent validity. The CFA further revealed that MSV < AVE, ASV < AVE; the square root of AVE was greater than the correlation between subscales, and the scale had discriminant validity [27, 28]. These two results show that the items in the scale are highly correlated with the subscales they are under and with the other items under the same factor and that the items have a low or no relationship with the other subscales and items. This result revealed that the items only measure the construct they are expected to measure without confusing them with other concepts, and that the scale has a strong factor structure [27, 28]. Convergent and discriminant validity analyses confirmed that the scale could successfully identify children with adequate and inadequate self-management from the parents' perspective. This indicates that the scale is a reliable and valid tool for assessing various aspects of self-management in children with T1DM.

After the construct validity of the scale was ensured, reliability analyses were performed. First, Cronbach's alpha was evaluated for internal consistency. The Cronbach's alpha coefficient indicates whether items measure similar characteristics. This value is an indicator of homogeneity in a scale. In newly developed scales, Cronbach's alpha value is recommended to be greater than 0.80 for the whole scale and 0.70 for the subscales [22, 26, 29, 30]. It is stated that the Cronbach's alpha values of scales are relatively high, and therefore, McDonald’s Omega coefficients should also be calculated. In this study, the Cronbach's alpha and McDonald's Omega coefficients of the scale were found to be above 0.80 for the whole scale and above 0.70 for the subscales, indicating that the scale has a very high reliability. The literature emphasizes that high Cronbach’s alpha and McDonald’s Omega coefficients indicate strong internal consistency, meaning that the items are compatible with each other and measure only the construct that is intended to be measured [21, 31–34]. This result shows that the scale is highly reliable. This also shows that all scale items are consistent with each other and measure the same conceptual structure.

In this study, item-total score correlation, item-subscale score correlation, and the correlation between the total scale score and the subscale scores were found to be positive, statistically significant and above 0.30. These results show that all the items of the scale have a sufficient level of correlation with the total score of their own subscales, the item reliability of the subscales is high, and the scale has a reliable and high internal consistency [21, 31–34].

In the study, split-half analysis was also performed. The literature suggests that in this analysis, the correlation between the two halves should be at least 0.70; the Cronbach alpha values of both halves should be greater than 0.70, and the Spearman-Brown and Guttman half coefficients should be greater than 0.80 [22, 26, 29, 30]. The results of the split-half analysis were found to be above the values recommended in the literature. These results showed that the scale has a high level of reliability.

Item-total score analysis shows whether the items in the scale measure the construct that is intended to be measured [21, 24]. It is recommended that the item-total score correlation should be at least 0.30. In this study, the item-total score correlations exceeded the values stated in the literature, demonstrating that the items were closely related to the overall scale. This indicates that the items were pertinent to the construct being measured, showed a homogeneous distribution, and exhibited high internal consistency. Measurement reliability is crucial in assessing changes in self-management over time in children with T1DM. The scale demonstrated the ability to detect similar results across different conditions, particularly in children whose self-management scores remained stable. Consequently, the scale was shown to produce both accurate and consistent measurements.

In order to obtain accurate results in scales, there should be no response bias [21, 31–34]. The response bias analysis in this study showed that all the participants filled in the scale according to their own opinions and there was no bias that would affect the scale results [21, 31–34].

The scale, which was found to provide accurate and consistent measurements, can be used to evaluate children's self-management. According to the literature, self-management in children with T1DM is crucial for preventing acute and chronic complications. Effective T1DM management also significantly improves children's quality of life. Children with T1DM should adhere to a self-management plan that includes daily blood glucose monitoring, insulin injections, physical activity, and adequate and balanced nutrition to maintain blood glucose levels within the reference range and minimize the risk of complications [35]. Failure to maintain good metabolic control can result in acute complications, chronic complications, and even death [35–37].

Accurate and consistent determination of T1DM self-management is crucial for identifying the needs of children and preventing future complications. Using valid and reliable measurement tools is essential for this purpose. This study is significant because it introduces a scale that effectively assesses self-management in children with diabetes, contributing valuable insights to the literature.

There are several limitations of the study. The first limitation is the use of convenience sampling. However, it is thought that the sufficient sample size, the follow-up of patients from different regions of Turkey, the university hospital where the study was conducted, and the homogeneous distribution of the participants can reduce the problems related to generalizability. The second limitation is that the data were collected based on self-report. The results of the study are limited to the information provided by the parents. Finally, the study results are based solely on cross-sectional survey data obtained at a single point in time. It is recommended to confirm the results with longitudinal follow-up studies.

## Conclusion

The study revealed that the T1DM Self-management Scale for Parents is valid and reliable tool for assessing the level of self-management in parents of children with diabetes. This self-reported measurement tool evaluates how well parents manage their child’s T1DM. Using this scale, researchers can identify the factors influencing self-management levels and design interventional studies to address these factors. The scale also allows for monitoring and evaluating the impact of interventions on parents' diabetes self-management skills. Additionally, the scale can be adapted for different cultures, enabling cross-cultural comparison studies.

## Implications for practice

Validity and reliability studies of this highly accurate and consistent scale can be conducted for various age groups. The scale can be used to assess the self-management levels of children with diabetes and factors affecting the level of self-management. Interventional studies can be designed to address factors that decrease self-management. Since the scale was developed using guidelines from institutions such as the ADA and WHO, it includes universal items regarding diabetes self-management. Therefore, researchers can adapt this scale to different societies and use it effectively in related studies.

We extend our gratitude to all the parents, experts, institutions, and their employees who participated in and contributed to this study. We also thank those who have contributed to the literature that provided the foundation for our research.

## Data Availability

Data may be requested from the authors by explaining the rationale.
